# Unusual Presentation of Aggressive Atypical Hemolytic Uremic Syndrome With Brugada Syndrome

**DOI:** 10.7759/cureus.66019

**Published:** 2024-08-02

**Authors:** Khalid Al Balushi, Abdullah Al Lawati, Issa Al Salmi, Ehab Mohammed, Abdulrahman Al Hadhrami, Naima Al Alawi, Khalfan Al-Shaaili

**Affiliations:** 1 College of Medicine and Health Sciences, Sultan Qaboos University, Muscat, OMN; 2 College of Medicine, Universal Scientific Education and Research Network (USERN), Tehran, IRN; 3 The Renal Medicine Department, The Royal Hospital, Muscat, OMN; 4 The Histopathology Department, The Royal Hospital, Muscat, OMN; 5 Nephrology Department, Nizwa Hospital, Nizwa, OMN

**Keywords:** complement system, eculizumab, thrombotic microangiopathy (tma), brugada sydrome, atypical hemolytic uremic syndrome

## Abstract

Hemolytic uremic syndrome (HUS) is part of a spectrum of disorders known as thrombotic microangiopathies. These disorders are characterized by giving rise to platelet microthrombi, which subsequently develop hemolytic anemia and thrombocytopenia. In HUS, the kidneys are destroyed, mainly due to damage to the renal blood vessels. HUS can be typical or atypical, depending on the cause, and can lead to significant mortality rates. We herein report an unusual case of atypical HUS in a 15-year-old female who presented with fatigue, abdominal pain with nausea and vomiting, loss of appetite, and urine discoloration. Further tests showed low platelets with significant anemia. She was diagnosed with atypical HUS after discovering that she had no previous bloody diarrhea episode with a negative *E. coli* strain, O157:H7, alongside valid ADAMTS13 activity. The diagnosis was confirmed by genetic testing, and a variant of uncertain significance was found in the CFH gene. The patient, therefore, was started on eculizumab, and a follow-up was done once or twice a month through blood testing. She showed significant improvement. Due to non-compliance with the eculizumab treatment, the patient showed deterioration numerous times. A kidney biopsy was subsequently done, showing signs of acute to chronic thrombotic microangiopathy with moderate tubular atrophy and interstitial fibrosis. After many hemodialysis and plasma exchange sessions and being put on several treatments, such as prednisolone and rituximab, the patient faced death after one year.

## Introduction

Thrombotic microangiopathies are disorders characterized by hemolytic anemia, thrombocytopenia, and ischemic tissue injury resulting from platelet microthrombi [1,2]. These disorders can be classified into two main categories, which are thrombotic thrombocytopenic purpura (TTP) and hemolytic uremic syndrome (HUS). HUS is a thrombotic microangiopathy that commonly affects the kidney, while TTP primarily occurs in adults and presents with a pentad of fever, neurologic abnormalities, thrombocytopenia, microangiopathic hemolytic anemia, and impaired renal function. It results in impaired renal function and is typically caused by bacterial exotoxins such as the Shiga-like toxin from enterohemorrhagic *E. coli* (STEC-HUS) strain O157:H7 and the Shiga toxin produced by *Shigella dysenteries* [1,3]. The latter is often accompanied by a diarrheal illness (usually bloody) lasting 5-10 days that precedes the HUS symptoms [1]. A local study found that the HUS population was mainly due to Shiga toxin-producing *Escherichia coli* (STEC). It showed that the HUS population was young and primarily male, and only 25% had known medical comorbidities at the time of presentation. Also, the majority presented with acute kidney injury (AKI) requiring dialysis, of which peritoneal dialysis (PD) was the mainstay of therapy. The duration of renal replacement (RRT) and recovery time was almost a month [1]. 

There are different types of HUS. Atypical HUS (aHUS) represents a thrombotic microangiopathy characterized by uncontrolled complement activation, usually unmasked by a complement-amplifying condition such as infections, surgery, trauma, autoimmune diseases, transplantation, and certain medications. It differs in pathophysiology from the typical HUS as bacterial toxins are not the primary cause of endothelial cell damage. AHUS is related either to a genetic deficiency in one or more soluble and/or membrane-bound complement regulatory proteins, usually Complement Factor H (CFH), or an acquired defect producing autoantibodies to complement-related factors, resulting in uncontrolled complement activation in the alternative pathway and thus inflammation of different blood vessels in the body [4,5]. A study conducted in Italy revealed the mean prevalence of HUS among children to be 6.3 cases per million children < 18. Atypical HUS accounted for 11.9% of the cases, with a mortality rate of 8.3% compared to 3.4% in STEC-HUS [6]. We present a case of a young female patient suffering from an aggressive a-HUS with manifestations in various systems of the body, with sudden, unexpected death at home.

## Case presentation

A 15-year-old female presented to the Emergency Department of our tertiary hospital in July 2022 with complaints of fatigue, abdominal pain, nausea, vomiting, loss of appetite, and urine discoloration. She denied any episodes of fever or diarrhea but appeared pale and mildly jaundiced upon clinical examination. There was no history of neurological symptoms or deficits; her brother was diagnosed with aHUS and received treatment with Eculizumab for six years, then fully recovered. Various laboratory tests were ordered, as shown in Table 1, revealing that low hemoglobin was also suspected but was ruled out with further testing. levels (Hb) and low platelets. Consequently, she received one unit of packed red blood cells and was admitted with the impression of microangiopathic hemolytic anemia (MAHA) and thrombocytopenia. Other differential diagnoses were considered, including thrombotic thrombocytopenic purpura (TTP) and typical/atypical hemolytic uremic syndrome (HUS). Dengue fever was also suspected but was ruled out with further testing. Additional laboratory findings indicated microcytic hypochromic anemia with nucleated red blood cells (RBCs). Furthermore, the blood film displayed polychromatic RBCs with schistocytes, and white blood cells (WBCs) showed a left shift with band forms but no blasts. Platelets were low, with large, giant platelets observed. Genetic testing was conducted, revealing a pathological variant in the G6PD gene, confirming a genetic diagnosis of X-linked hemolytic anemia due to G6PD deficiency. Additionally, another variant of uncertain significance was found in the CFH gene, potentially indicating autosomal dominant atypical HUS type, along with a variant of uncertain significance in the GPD1L gene, suggesting autosomal dominant Brugada syndrome type 2. 

**Table 1:** History of eculizumab administration CBC with blood film results

Date (Eculizumab dose)	Complete Blood Count (CBC) Panel					
	Hb (g/dL)	Platelets (mm^3)	WBC (mm^3)	Neutrophils (%)	Lymphocytes (%)	Blood film
19/07/22	3.4	48	5	3.6	0.8	Microcytic hypochromic red blood cells with many red cell fragments.
03/08/22 (900)	8.4	122	10	7.9	1.3	Dysmorphic blood picture, mild microcytic hypochromic.
10/08/22 (900)	6.5	100	6.6	6	0.4	-
17/08/22 (900)	7.6	88	10.7	7.7	2.9	Marked microcytic hypochromic with few fragmented cells.
07/09/22(900)	7.2	147	5.6	4.1	0.8	Dysmorphic blood picture with mild microcytic hypochromic.
21/09/22 (1200)	8.6	228	4.4	2.6	1.1	Mild microcytic hypochromic with few teardrop cells.
04/10/22 (1200)	9.6	385	8.9	6.3	1.5	Mild microcytic hypochromic with many burr cells.
20/10/22 (1200)	10.8	357	12.9	10.4	1.8	-
06/11/22 (900)	11.4	431	10.3	7.3	2	-
20/11/22 (900)	11.7	172	11.3	8.7	1.7	-
13/01/23 (1200)	7.2	59	12	9.5	1.6	Moderate microcytic hypochromic, with many fragmented cells.
26/01/23 (1200)	7.2	245	12.2	10.6	0.8	Dysmorphic blood picture with mild microcytic hypochromic.
09/02/23 (1200)	5.3	53	14.7	12.2	1.7	Moderate microcytic hypochromic, with moderate polychromasia.
23/02/23 (900)	6.5	153	9.2	7.2	1	Mild microcytic hypochromic with few fragmented cells.
09/03/23 (900)	7.4	188	13.7	11.1	1.5	-
26/03/23 (900)	7.5	198	12.7	11.6	0.7	-
10/04/23 (900)	9	271	17	15.8	0.6	-
26/04/23 (900)	8.4	264	12.7	10.8	1	-

On the sixth day of admission, she commenced plasma exchange via the femoral line, resulting in an anaphylactic reaction with three episodes of seizures. A computed tomography (CT) scan of the brain revealed bilateral high parietal and right frontal hypodensities consistent with posterior reversible encephalopathy syndrome (PRES) resulting from uncontrolled hypertension, which was thought to be the cause of the seizures. Subsequently, she was intubated after the seizure and initiated on levetiracetam. On the same day, she developed acute renal failure and underwent one session of hemodialysis. Despite treatment, her blood pressure remained uncontrolled, leading to the initiation of antihypertensive medication, nifedipine, during her admission. Later, the patient received five sessions of plasma exchange and commenced renal replacement therapy. Additionally, she received pulse methylprednisolone for three days, followed by oral prednisolone at 1 mg/kg. Furthermore, a dose of 900 mg per week of eculizumab was initiated. During the evaluation of the patient for possible TTP, blood was collected on the sixth day for genetic testing of the ADAMS 13 activity. Two months later, the ADAMS 13 score was found to be 48% (50-150% is the normal baseline), effectively ruling out TTP. With eculizumab treatment, her renal function and hemolytic parameters significantly improved, leading to an increase in the dose to 1200 mg per week, as shown in Table 1. The plan was to continue eculizumab until the genetic workup for aHUS was completed, including factor H (CFH) mutation analysis and whole exome sequencing. Hepatitis testing was done to rule out hepatitis based on her symptoms. A month later, her hepatitis serology screen showed positive antibodies to hepatitis B core antigen (anti-HBc) and surface antigen antibodies (anti-HBs). The hepatitis infection was thought to be caused by her long hospital stay. Consequently, she started on entecavir, 0.5 mg once daily, which was to be continued for at least a year after discontinuation of immunosuppression.

In January 2023, six months after her initial visit, she presented again with new-onset lower abdominal pain associated with nausea, vomiting, and coughing. There was no fever or change in bowel habits, but she had recently returned from overseas travel, which caused her to miss the 12th dose of eculizumab, therefore delaying her dose by two weeks. Laboratory findings revealed grossly deranged renal function, suggestive of acute kidney injury (AKI), and severe anemia. She had low hemoglobin (Hb 4.5) with increased hemolytic markers and elevated urea, indicating a possible TMA relapse. Apart from an elevated blood pressure (170/110) and lower abdominal tenderness, her examination was unremarkable. Therefore, given her presentation, a femoral vein catheter was inserted, and hemodialysis was initiated with leucocyte-depleted packed red blood cells (3 units) transfused. The following day, she developed bloody, loose motions, and clostridium toxin A&B tested positive. After treatment for clostridium-related diarrhea, eculizumab was resumed, and the patient underwent a total of five sessions of plasma exchange. Subsequently, the patient improved and was discharged home on January 26, 2023, but remained dialysis dependent. However, the next day after discharge (January 27, 2023), she presented to the emergency room with hypertensive pulmonary edema and shortness of breath (SOB). She was also febrile, with high inflammatory markers and neutrophilic leukocytosis. Urgent hemodialysis and ultrafiltration were initiated, and she was started on piperacillin/tazobactam. The subsequent day, a positive blood culture from a peripheral vein indicated the development of catheter-related bloodstream infection (CRBSI) for gram-positive cocci. Consequently, the jugular venous catheter was removed, and she was started on vancomycin in addition to piperacillin and tazobactam. An echocardiogram revealed no vegetation indicative of bacterial endocarditis.

On February 1, 2023, the patient was discharged home. Exactly a week later, she once again presented to the ED with SOB and hemoptysis. Her blood pressure was 186/96 mmHg, and her physical examination revealed bilateral basal crepitation and orthopnea. Laboratory tests indicated a very low Hb of 5 g/dL. Therefore, she was admitted and received one unit of leucocyte-depleted pRBC and her 14th dose of eculizumab. The subsequent day, her Hb dropped to 3.8, her platelets were 34, and her WBC count was 7.6, prompting a transfusion with three more PRBCs. The blood film showed microcytic hypochromic anemia, moderate schistocytes (2-3%), slight neutrophilia with left shift, and thrombocytopenia. Despite transfusions, her Hb levels continued to drop over the following week, leading to positive occult blood testing. She underwent esophagogastroduodenoscopy (OGD), revealing features of chronic gastritis. Consequently, she was discharged on omeprazole and antacids, with a follow-up appointment scheduled for her next eculizumab dose a week later. The patient remained on regular follow-up and received her eculizumab doses regularly. Her last regular hemodialysis session was in February 2023. Due to her renal function improvement, she was kept off dialysis. On 26/04/2023, her lab values indicated a serum creatinine of 120 umol/L, blood urea of 6.8 mmol/L, Hb of 8.4 g/L, platelet count of 264, WBCs of 12.7, and CRP < 4. However, the patient missed her regular follow-up, and we later learned that she unfortunately passed away at home, possibly secondary to sudden cardiac arrhythmias while asleep, which was confirmed by an autopsy.

**Figure 1:**
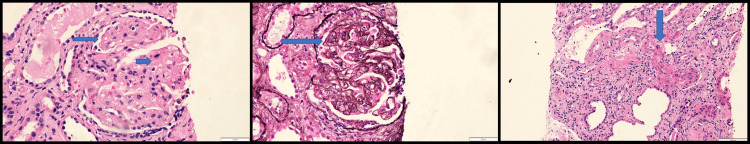
Microscopy images of the kidney glomerular basement membrane and blood vessels at different magnification powers Left to right: Left: H&E, X40: This glomerulus appears bloodless with mesangiolysis (small arrow). The capillary walls are thick and contain swollen endothelium (long arrow). Middle: Jones methenamine silver stain, x40: The capillary walls are corrugated and show segmental double contouring (arrow), indicating chronic TMA. Right: H&E, X 20: Interlobular artery showing acute TMA with fragmented red blood cells in the wall (arrow).

**Figure 2:**
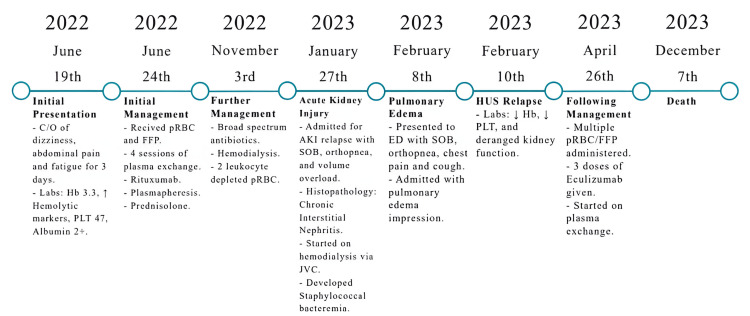
Stepwise progress of the patient from the initial encounter to her sudden, unexpected death

Tables 2-4 show the various laboratory investigation results over the course of the treatment period, including hemolytic markers (Table 2), urea and electrolytes (Table 3), and liver function tests (Table 4). The kidney histopathology report also describes fibrosis, inflammation, and scarring levels among different parts of the biopsy tissues. Figure 1 is a collage of the microscopy images of the kidney glomerular basement membrane and blood vessels at different magnification powers. Figure 2 illustrates the stepwise progression of the patient from her initial admission until her demise. 

**Table 2:** History of hemolytic markers values

Date (Eculizumab dose)	Hemolytic Markers			
	Absolute reticulocyte count (cells/mm^3)	Lactate dehydrogenase (U/L)	Haptoglobin (mg/dL)	C-reactive protein (mg/L)
19/07/22	460	1521	<10.00	22
03/08/22 (900)	168	493	<10.00	4
10/08/22 (900)	-	-	<10.00	<4.00
17/08/22 (900)	227	440	<10.00	6
07/09/22(900)	-	335	<10.00	-
21/09/22 (1200)	-	333	450	14
04/10/22 (1200)	-	335	960	20
20/10/22 (1200)	-	198	765	<4.00
06/11/22 (900)	-	177	837	<4.00
20/11/22 (900)	-	-	<10.00	<4.00
13/01/23 (1200)	196	468	<10.00	27
26/01/23 (1200)	68.5	281	330	46
09/02/23 (1200)	121	389	<10.00	8
23/02/23 (900)	275	438	<10.00	<4.00
09/03/23 (900)	-	357	<10.00	4
26/03/23 (900)	-	451	13	<4.00
10/04/23 (900)	-	-	<10.00	<4.00
26/04/23 (900)	-	-	<10.00	<4.00

**Table 3:** History of urea and electrolytes lab values

Date (Eculizumab dose)	Urea & Electrolytes					
	Sodium (mmol/L)	Potassium (mmol/L)	Carbon dioxide (mmHg)	Chloride (mmol/L)	Urea (mg/dL)	Creatinine (µmol/L)
19/07/22	134	3.2	24	98	8	98
03/08/22 (900)	141	4	32	105	18.9	275
10/08/22 (900)	137	4.2	22	104	16.4	251
17/08/22 (900)	140	3.6	21	108	10.7	170
07/09/22(900)	141	3.9	22	108	4.8	141
21/09/22 (1200)	142	3.5	26	107	3	103
04/10/22 (1200)	141	4	25	105	2.2	100
20/10/22 (1200)	140	3.9	24	106	3.1	95
06/11/22 (900)	139	3.9	22	107	3.5	94
20/11/22 (900)	139	4.1	26	105	4.1	92
13/01/23 (1200)	130	3.6	22	95	28.5	757
26/01/23 (1200)	136	4.3	24	103	30.8	326
09/02/23 (1200)	141	3.9	23	105	32.1	283
23/02/23 (900)	138	3	24	101	17.2	187
09/03/23 (900)	139	3.5	25	102	8.8	148
26/03/23 (900)	138	3.5	25	104	7.2	140
10/04/23 (900)	139	4	24	104	7.7	128
26/04/23 (900)	139	3.1	25	103	6.8	120

**Table 4:** History of liver function test values

Date (Eculizumab dose)	Liver function test					
	Total bilirubin (mg/dL)	Alanine transaminase (U/L)	Alkaline phosphatase (U/L)	Total protein (g/L)	Albumin (g/L)	Globulin gap (g/L)
19/07/22	16	17	75	57	26	31
03/08/22 (900)	12	64	68	52	28	24
10/08/22 (900)	9	15	65	49	27	22
17/08/22 (900)	7	13	66	56	29	27
07/09/22(900)	5	15	72	58	33	25
21/09/22 (1200)	4	11	73	51	26	25
04/10/22 (1200)	<3	12	71	53	25	28
20/10/22 (1200)	-	-	-	-	-	-
06/11/22 (900)	-	-	-	-	-	-
20/11/22 (900)	7	14	72	63	35	28
13/01/23 (1200)	4	<7	52	41	19	22
26/01/23 (1200)	9	18	59	44	30	14
09/02/23 (1200)	12	34	53	54	35	19
23/02/23 (900)	13	22	66	60	38	22
09/03/23 (900)	9	11	83	55	36	19
26/03/23 (900)	7	13	70	63	38	25
10/04/23 (900)	7	9	89	62	36	26
26/04/23 (900)	9	9	73	60	36	24

The kidney biopsy histopathology report revealed several notable findings. Out of approximately six glomeruli, two were sclerotic, and one had a segmental scar. The rest showed varying degrees of ischemic changes, such as collapsed tufts and mesangiolysis. One glomerulus had thickened capillary walls and segmental double contouring with red blood cells inside the membrane. There was no evidence of thrombosis, necrosis, or crescents. The tubules were moderately injured, with a flattened lining epithelium, and about 30% of the cortical parenchyma showed moderate atrophy. In the interstitium, moderate fibrosis affected roughly 30% of the cortex and was accompanied by inflammatory infiltrates of lymphocytes and plasma cells. A section of an interlobular artery in the biopsy showed mild thickening of the intima with duplication of the internal elastic lamina. Additionally, three arterioles displayed endothelial swelling, fibrinoid necrosis in their walls, and fragmented red blood cells. Immunofluorescence did not detect any glomeruli, and while electron microscopy was not performed, one obsolete glomerulus was present. The diagnosis from these findings is acute chronic thrombotic microangiopathy, along with moderate tubular atrophy and interstitial fibrosis.

## Discussion

This is the first case reported to have two very rare disorders, aHUS and Brugada syndrome. HUS is one of the microangiopathic hemolytic anemia diseases that are known to be non-immune and Coombs-negative. The typical cause of HUS is predominantly bacterial, and pathophysiology can be explained by the toxins produced by bacteria that damage the vascular endothelium. As a result, loose strands of platelets and fibrin deposit in the affected endothelium, damaging the RBCs and platelets that pass by, producing features of thrombocytopenia and hemolytic anemia. HUS can rarely develop a genetic component differing in the pathophysiology from the typical HUS, thus called atypical HUS (aHUS) [1].

Atypical HUS, also known as diarrhea-negative HUS, often results from environmental and genetic factors. Mutations in the gene CFH are present in 30% of cases of atypical HUS, and this gene is involved in regulating the complement system present in the body. Thus, mutations in this gene often result in the overactivity of the alternative pathway of the complement system, resulting in the inflammation of blood vessels, particularly in the kidneys, causing inflammation and the formation of clots, therefore causing kidney damage and, in many cases, kidney failure and ESRD [7]. A study from Oman revealed that the prevalence of atypical HUS in a population involving 36 patients was six (16.67%); most of the patients diagnosed with HUS were males with a mean age of 10 [1]. A 10-year retrospective study in Europe revealed that nine individuals out of 47 had aHUS during this period; 56% were males, and all were less than 10 years of age. Among those, the etiology varied from infectious causes such as pneumococcal infection to genetic causes [6-8]. According to a study done in the USA, it was revealed that patients with aHUS have more extended hospital stays and are more likely to receive dialysis compared to typical HUS. The mortality rate among aHUS patients differs from country to country, at 2.78% in Oman compared to 8.3% in Italy [1,6].

Unlike individuals with typical HUS, who usually recover from the life-threatening initial episode and usually respond well to supportive treatment, individuals with aHUS are much more likely to develop chronic serious complications such as severe high blood pressure and kidney dysfunction [4,7,9]. In this present case, the young lady presented with multi-system findings involving the kidneys, pulmonary, gastrointestinal, and cardiovascular systems, for which she underwent many investigations to rule out possible complications. The present case died suddenly while asleep due to very rare disorders that include aHUS and Brugada syndrome, and both have a high predisposition for sudden severe cardiac outcomes. Brugada syndrome was named after Spanish cardiologists Pedro Brugada and Josep Brugada, who reported it as a distinct clinical syndrome in 1992. It is an inherited disease and a potentially life-threatening disorder [10]. It is a channelopathy with an increased risk of fatal arrhythmias that begin in the ventricles with a right bundle branch block and ST segment elevations in the right precordial leads. Screening family members with ECG and genetic testing is recommended to identify the positive cases, even if they are asymptomatic. Genetic testing for a mutation in SCN5A usually confirms the diagnosis [11]. Cardiovascular complications may occur in up to 10% of these populations. In aHUS, sudden death may ensue as a result of microangiopathic injury in the coronary microvasculature [12]. In Brugada syndrome, a sudden acute ST-elevation myocardial infarction may occur without any specific traditional cardiac risk factors present in these patients. The causes of these STEMIs include coronary vasospasm, coronary dissection or aneurysm, and coronary embolism [13]. In addition, high-output cardiac dysfunction from anemia and microangiopathic damage in the cardiac vasculature causes unpredictable symptoms ranging from myocardial infarction and cardiomyopathy to acute decompensated heart failure [14,15].

## Conclusions

This is the first case reporting a rare outcome of sudden death in a patient with two rare disorders, aHUS and Brugada syndrome. Atypical HUS is a variable disease that can result in multi-systemic complications if not managed adequately. Thus, a prompt diagnosis and proper intervention are of utmost importance to reduce cardiovascular risks and avoid deterioration in the patient’s condition.
